# Epidemiology and diagnosis technologies of human metapneumovirus in China: a mini review

**DOI:** 10.1186/s12985-024-02327-9

**Published:** 2024-03-07

**Authors:** Yuan Feng, Tao He, Bo Zhang, Haibin Yuan, Yinfei Zhou

**Affiliations:** 1 Xiangtan Central Hospital, Xiangtan, 411100 Hunan China; 2 Xiangtan Maternal and Child Health Hospital, Xiangtan, 411100 China

**Keywords:** Human metapneumovirus, Epidemiological characteristics, Genotype, Diagnosis methods, China

## Abstract

Human metapneumovirus (HMPV) is a newly identified pathogen causing acute respiratory tract infections in young infants worldwide. Since the initial document of HMPV infection in China in 2003, Chinese scientists have made lots of efforts to prevent and control this disease, including developing diagnosis methods, vaccines and antiviral agents against HMPV, as well as conducting epidemiological investigations. However, effective vaccines or special antiviral agents against HMPV are currently not approved, thus developing early diagnosis methods and knowing its epidemiological characteristics will be beneficial for HMPV control. Here, we summarized current research focused on the epidemiological characteristics of HMPV in China and its available detection methods, which will be beneficial to increase the public awareness and disease control in the future.

**Supplementary Information:**

The online version contains supplementary material available at 10.1186/s12985-024-02327-9.

## Introduction

Acute respiratory tract infections (ARTIs) are responsible for the high morbidity and mortality of human beings, which have been considered one of the most significant factors threatening public health worldwide [1]. Moreover, ARTIs often result in lower respiratory tract infections (LRTIs) in clinical, which are responsible for millions of deaths worldwide [2]. Numerous viruses, such as the coronavirus, influenza virus, and human respiratory syncytial virus (hRSV), and others, are responsible for the incidence of ARTIs. In 2001, a research team in the Netherlands first discovered a novel virus associated with ARTIs, which was named human metapneumovirus (HMPV) [3]. Subsequently, the prevalence of HMPV has been documented in many countries and is considered a leading cause of ARTIs worldwide owing to the limited prevention and control measures [4, 5]. The clinical symptoms of HMPV infection in infants, elderly individuals, and immunocompromised individuals are mainly characterized by fever, cough, and gasp for breath, etc.,[6], which are similar to those of other common ARTIs-related viruses’ infection, including human bocavirus and hRSV [7].

HMPV is an enveloped, non-segmented, negative-stranded virus that belongs to the subfamily *Pneumovirinae* [8]. The HMPV genome (nearly 13 kb) contains eight genes that encode nine proteins, namely nucleoprotein (N), phosphoprotein (P), matrix protein (M), fusion protein (F), matrix-2 proteins (M2-1 and M2-2), small hydrophobic (SH) protein, glycoprotein (G), and large (L) polymerase protein [9]. According to the genetic features of the F and G genes, HMPV strains prevalent worldwide could be classified into four genotypes (A1, A2, B1 and B2), and further divided into six lineages (A1, A2a, A2b, A2c, B1, and B2) [10, 11]. Notably, co-prevalence of several sub-genotypes or sub-lineages of HMPV has been frequently reported in the areas under investigation, while the thorough relationship between the disease severity and HMPV genotype is yet unclear [12, 13].

HMPV has been widely prevalent worldwide and continuously brings a significant medical burden to the local area, as effective vaccine or antiviral drug for treating or preventing HMPV infection is not licensed [14]. For instance, HMPV infection is associated with nearly one million outpatient clinic visits with clinical symptoms of ARTIs, and the disease caused by HMPV infection poses huge threats to the health of more than 86% of children aged below 5 years worldwide [15, 16]. Thus, a comprehensive understanding of the epidemiological characteristics, and detection approaches targeting HMPV would be beneficial for preventing this disease. This review provides updated information on HMPV in China, mainly focusing on its diagnosis approaches, prevalence, and genotype characteristics.

### Current molecular diagnosis methods for HMPV detection

Early diagnosis of HMPV infection will help to develop effective measures against the disease, such as limiting the outbreak and providing timely care for the patients. Since the highly conserved F protein amino acid sequences of HMPV and RSV [17], limited serological technologies were developed for detecting HMPV-specific antibodies [18–21]. Thus a variety of molecular diagnosis methods, probing viral nucleic acids, have been invented for HMPV molecular detection, which mainly include the reverse transcription polymerase chain reaction (RT-PCR), real-time quantitative reverse transcription polymerase chain reaction (RT-qPCR), and reverse transcription loop-mediated isothermal amplification (RT-LAMP), etc., (as summarized in Table 1).

**Table 1:** Summary of HMPV molecular diagnostic approaches and their characteristics

Methods	Operation time	Limit of detection	Experimental cost	Characteristics
RT-PCR [24, 25]	3–5 h	1000 copies/reaction	Low	RT-PCR has been widely used for performing epidemiological investigation of HMPV, while which requires complex instruments and trained workers
RT-qPCR [27, 28]	1–3 h	10 ~ 100 copies/reaction	Low	RT-qPCR has been widely amplified in HMPV monitoring in clinical samples
LAMP [34, 35]	~ 1.5 h	< 10 copies/reaction	High	Advantages: High sensitivity and specificity, rapid diagnosis with simple reaction procedure, and constant temperature Disadvantages: High requirements of primers, high false positive rate, and high cost
RAA [38]	15 ~ 30 min	100 copies/reaction	High	Advantages: High sensitivity and specificity, and rapid diagnosis of virus infection Disadvantages: High cost and high positive rate
RT-RPA combined with CRISPR-Cas12a [45]	< 30 min	< 700 copies/reaction	High	Advantages: High sensitivity and specificity, and rapid diagnosis of virus infection Disadvantages: High cost and high positive rate
mNGS [48]	5 ~ 10 days	Not determined	High	Advantages: High sensitivity and specificity, and rapid diagnosis of unknown pathogens Disadvantages: High cost and time-consuming
Virus isolation [51]	3 ~ 4 days or more time	Not determined	High	Advantages: The “gold standard” for pathogen detection Disadvantages: High cost and time-consuming, and low isolation rate, and requires complex instruments and trained workers

### Molecular detection methods developed based on PCR

#### RT-PCR

In the past decades, RT-PCR assays have been widely applied for viral molecular detection, including HMPV. Generally, the genomic regions with high sequence homology of HMPV, such as the F and N genes, are employed as molecular markers for developing the RT-PCR methods, and these targeted regions can be used for genotype analysis [22, 23]. Li et al. developed a GenomeLab Gene Expression Profiler genetic analysis system (GeXP) based multiplex RT-PCR method for rapid and sensitive detection of sixteen ARTIs-related pathogens (including HMPV), and the detection limit of each virus was 1000 copies/reaction when all viral targets were added into the reaction [24].

Generally, RT-PCR assays for pathogen detection display lower sensitivity compared with RT-qPCR methods, which also need complex instruments [25]. Thus, RT-PCR methods have been less applied for HMPV-clinical detection in recent years.

#### RT-qPCR

RT-qPCR is a highly sensitive, accurate, and efficient technology, which has been widely used for detecting viral nucleic acids [25–28]. Usually, RT-qPCR methods provide higher sensitivity, and lower probability of contamination compared to the conventional RT-PCR methods, thus which is designed as the gold standard diagnostic approach [29]. A TaqMan-based RT-qPCR method developed by Lu et al*.* in 2008 showed a limited detection of 10 copies/μL, 19.62% (31/158) clinical samples were detected positive for HMPV using the established RT-qPCR method, while only 13.92% (22/158) of tested samples were confirmed positive using the conventional RT-PCR method [26]. Sugimoto et al*.* developed a duplex RT-qPCR assay for HMPV detection, which could identify and differentiate between HMPV A and B subgroups with high sensitivity (< 10 copies/reaction) [27].

Since multiple virus infections result in ARTIs, multiplex RT-qPCR assays have been developed for synchronously detecting HMPV and other ARTIs-related pathogens. You et al. established a reliable triple TaqMan-based RT-qPCR method for detecting HMPV, RSV, and the internal control gene (GAPDH), and the limit of detection of the established method was 100 copies/reaction of both HMPV and RSV [28]. Moreover, the multiplex one-tube nested RT-qPCR assay established by Feng et al*.* [30]*,* could differentiate RSV, human rhinovirus (HRV), and HMPV, and the analytical sensitivity of the developed method was 5 copies/reaction for all three pathogens.

In recent years, digital microfluidic (DMF) technology has been applied in various fields including pathogen diagnosis [31]. Huang et al*.* developed an RT-qPCR platform combined with DMF technology for multiple detection of eleven respiratory pathogens including HMPV [32]. The sensitivities of the off-chip and on-chip RT-qPCR methods for multiple pathogens detection were ≤ 150 copies/reaction and ≤ 120 copies/reaction, respectively [32].

### Nucleic acid isothermal amplification methods

#### LAMP

The LAMP technology, first invented by a Japanese team in 2000, is one of the most widely used isothermal methods in pathogen diagnosis [33]. Song et al*.* used the online LAMP primer design software Primer-Explorer V4 to design four pairs of primers targeting the M genes, to establish two RT-LAMP reactions for detecting and differentiating HMPV genotypes A and B, respectively [34]. The limit of detection (LOD) of the established method for HMPV genotypes A and B was 4.33 copies/μL and 3.53 copies/μL, respectively, which was 10 times more sensitive than the conventional RT-PCR methods [34]. Similarly, Wang et al. designed three pairs of primers targeting the N gene for HMPV detection. The established method showed a sensitivity level of < 10 copies/μL, which was more sensitive than the RT-PCR method [35].

Collectively, LAMP is performed in an isothermal environment (~ 65℃) with high amplification efficiency, which does not need complex equipment for thermal change. Meanwhile, the LAMP method displays higher specificity since two or three pair of primers are included in the reaction system, which can also improve the amplification efficiency [36]. Moreover, the detection results of the LAMP method can be observed and judged via the naked eyes, which further simplify the detection procedures [35, 36].

#### Recombinase-aided amplification (RAA)

RAA is a newly invented thermostatic amplification method, which can be performed with easy operation processes, simple equipment, and high amplification efficiency, and has been widely applied in field diagnosis of different pathogens [37]. Jiao et al*.* used the conservative region of the HMPV N gene to design primers for inventing the RT-RAA method, the LOD of the established method was 100 copies/μL, which was significantly lower than the commercial RT-qPCR method [38]. Moreover, the RT-RAA reaction system was set 39℃ for 15 min, which needed less running time compared with the RT-qPCR method [38].

#### CRISPR-Cas12a

Clustered regularly interspaced short palindromic repeat (CRISPR) and CRISPR-associated proteins (Cas) have been widely applied in gene editing areas [39]. According to the genetic characteristics of the Cas gene in prokaryotes, the Cas protein families have been divided into three types [40]. Among these, the CRISPR-Cas12a system is considered a powerful tool for detecting nucleic acids in vitro [41]. In addition, the sensitivity and robustness of the CRISPR-Cas12a detection system could be greatly improved by combining with simultaneously reverse transcription and isothermal amplification technologies, such as LAMP [42], RAA [43], and recombinase polymerase amplification (RPA) [44]. Moreover, the CRISPR-Cas12a detection result can be observed via the naked eye when combined with lateral flow (LF), thus the established technology might be applied in these point-of-need places, such as airports and customs centers [45].

Qian et al*.* successfully established an HMPV RNA detection technique via combining RT-RPA with the CRISPR-Cas12a system. Briefly, the HMPV N gene was amplified via RT-RPA, the products were detected via CTISPR-Cas12a combined with LF [45]. Overall, the RT-RPA combined with Cas12a-based LF assay could be completed within 30 min, with an LOD of < 700 copies/mL [45]. Moreover, the established RT-RPA-Cas12a-LF method showed 96.4% detection coincidence with the quantitative RT-PCR assay, indicating that it would be an alternative tool for HMPV diagnosis without specialized equipment [45].

#### Metagenomic next-generation sequencing (mNGS)

mNGS is an emerging high-throughput diagnostic method that has been widely employed for virus genome sequencing, identification of novel pathogens, and other research [46]. When novel pathogens emerge, traditional detection methods (such as RT-PCR and RT-qPCR) might not be able to identify these new pathogens, while the mNGS can identify unknown or emerging pathogens [47]. Moreover, mNGS can be also used to amplify the whole viral genome with high accuracy, while the RNA genome must be reverse transcripted into cDNA before sequencing using Illumina and Sanger sequencing [37]. Xu et al*.* used the nanopore metagenomic sequencing technology to investigate the nosocomial transmission of HMPV among hematology patients, this method successfully generated HMPV reads of 80% (20/25) samples and obtained the complete HMPV genomes from 15 samples [48]. These data indicated that this technology can be used for diagnosing HMPV infection, while the sensitivity should be further improved [48].

#### Virus isolation

Virus isolation from the clinical samples is regarded as a gold standard for pathogen diagnosis, which is the initial step to developing vaccines and investigating viral pathogenesis [49]. Usually, the human Chang conjunctiva cell line (clone 1-5C4), LLC-MK2 cell line, and feline kidney CRFK cell line are suitable for HMPV isolation, and the LLC-MK2 cell line was more frequently used in HMPV isolation and cultivation [50, 51].

The procedures of virus isolation mainly include two steps: 1) the supernatants of the HMPV-positive sample are incubated with cell monolayer; 2) after 48–96 h of post-infection, the HMPV antigens or nucleic acids could be detected via serological or molecular detection methods. As HMPV grows slowly in cell lines, and cytopathic effects are rarely observed in HMPV-cell lines, subsequent serological/molecular assays are frequently used to detect HMPV antigens/nucleic acids [51].

#### Current epidemiology of HMPV in China

**Fig. 1:**
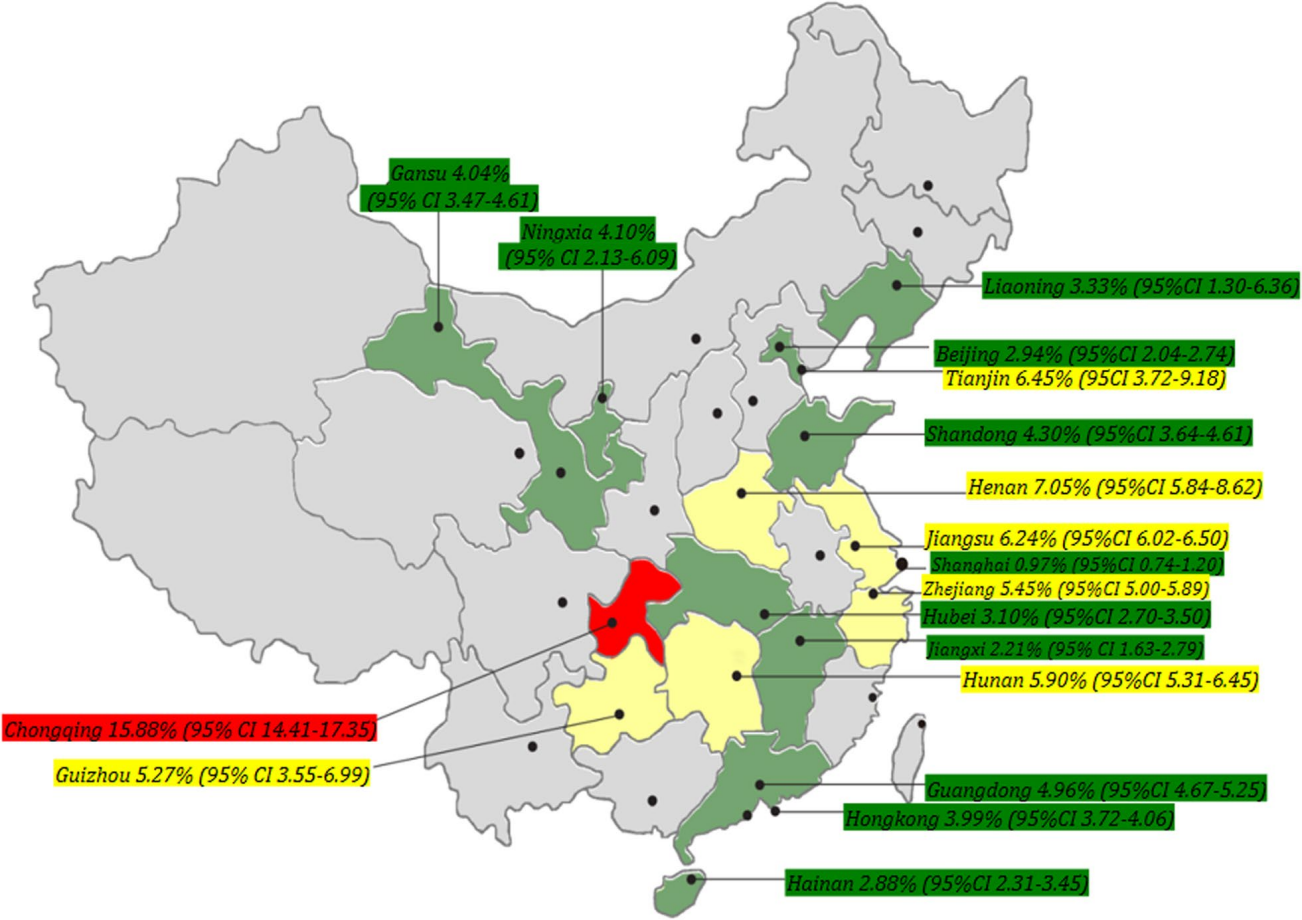
Current epidemiology of HMPV in China. results of 56 representative studies investigating the epidemiological characteristics of HMPV in human populations across distinct regions covering 18 provinces or regions. These studies were collected from the English and Chinese databases from January 1, 2003, to December 31, 2023. Data of these provinces or regions marked with white were not available

Since the first document of HMPV outbreaks in China in 2003 [52], the potential threats to public health have attracted widespread attention, thus epidemiological investigations have been frequently performed to monitor the prevalence of HMPV in China. To offer comprehensive and updated information on these, a total of 56 representative studies focusing on HMPV epidemiology across different regions in China were collected from the Chinese database and English database, the research was reported from January 1, 2003, to December 31, 2023 (Fig. 1). the detailed information of these research were shown in Supplementary Table 1.

#### Risk factors associated with HMPV epidemiology in China

A total of 188,104 clinical samples and 8846 HMPV-positive cases are included in this review, which yields the average positive rate of 4.70% (95% CI 4.61–4.80). In detail, the prevalence of HMPV among patients with acute respiratory illness from different provinces/regions ranged from 0.97% (95% CI 0.74–1.20) to 15.88% (95% CI 14.41–17.35). The highest prevalence of HMPV was observed in Chongqing city (15.88%, 95% CI 14.41–17.35), Henan (7.05%, 95% CI 5.48–8.62), Tianjin (6.45%, 95% CI 3.72–9.18), and Jiangsu (6.26%, 95% CI 6.02–6.50), while Shanghai, Jiangxi, Hainan, and Beijing showed the lowest prevalence, with < 3.0%.

Next, the potential risk factors in subgroups associated with HMPV infection, including gender, age, season, case type, and others, were estimated in this study. As shown in Table 2, in the age subgroups, the detection rate of HMPV infection among patients aged > 60 months (1.47%, 95% CI 1.30–1.64) was significantly lower than other age subgroups, while the prevalence of HMPV infection among in other age subgroups (< 6 months, 6–12 months, 12–36 months, and 36–60 months) showed no significant difference. Among different seasons, the highest positive rate of HMPV infection among patients was detected in spring (12.13%), followed by winter (3.56%), while the prevalence of which in summer (1.28%) and autumn (1.32%) showed no significant difference.

**Table 2:** Pooled molecular prevalence of HMPV infection in China

		No. of studies	No. of tested samples	No. of positive samples	Heterogeneity			% (95 CI)
					χ^2^	*p*-value	I^2^ (%)	
Region	Guizhou province	2	645	34	0.5	0.47	0	5.27% (3.55–6.99)
	Chongqing city	3	2374	337	460.8	< 0.01	99.6%	15.88% (14.41–17.35)
	Hongkong	4	20,917	835	19.6	< 0.01	84.7%	3.99% (3.72–4.26)
	Liaoning	1	300	10	1.2	0.27	–	3.33% (1.30–5.36)
	Beijing city	9	26,396	777	159.2	< 0.01	95.0%	2.94% (2.04–2.74)
	Tianjin city	1	310	20	2.2	0.14	–	6.45% (3.72–9.18)
	Ningxia province	1	390	16	0.3	0.59	–	4.10% (2.13–6.07)
	Gansu province	4	4629	187	4.1	0.04	26.8%	4.04% (3.47–4.61)
	Shandong province	2	3671	158	1.1	0.29	0.9%	4.30% (3.64–4.96)
	Jiangsu province	8	37,637	2355	157.8	< 0.01	95.6%	6.26% (6.02–6.50)
	Zhejiang province	5	9846	537	47.2	< 0.01	95.8%	5.45% (5.00–5.89)
	Shanghai city	3	6908	67	210.4	< 0.01	98.6%	0.97% (0.74–1.20)
	Guangdong province	7	22,050	1094	3.5	0.06	–	4.96% (4.67–5.25)
	Hainan province	1	243	7	1.8	0.19	–	2.88% (2.31–3.45)
	Jiangxi province	1	2438	54	32.9	< 0.01	–	2.21% (1.63–2.79)
	Hubei province	2	7248	225	38.9	< 0.01	97.4%	3.10% (2.70–3.50)
	Hunan province	3	6962	411	22.2	< 0.01	91.0%	5.90% (5.31–6.45)
	Henan province	1	1021	72	12.8	< 0.01	–	7.05% (5.48–8.62)
Gender	Female	16	35,669	1823	62.2	< 0.01	75.9%	5.11% (4.88–5.34)
	Male	16	57,468	2001	37.2	< 0.01	59.7%	3.48% (3.33–3.62)
Age	< 6 months	8	12,991	582	30.8	< 0.01	77.3%	4.48% (4.12–4.83)
	6 –12 months	8	8771	371	12.4	< 0.01	43.5%	4.23% (3.81–4.65)
	12 –36 months	10	18,248	766	20.8	< 0.01	56.7%	4.20% (3.91–4.49)
	36–60 months	11	16,454	634	5.1	0.02	–	3.85% (3.56–4.14)
	> 60 months	11	18,790	276	205.9	< 0.01	95.1%	1.47% (1.30–1.64)
Season	Spring (Mar–May)	5	3076	373	284.1	< 0.01	98.6%	12.13% (10.98–13.28)
	Summer (Jun–Aug)	5	3051	39	61.8	< 0.01	93.5%	1.28% (0.88–1.68)
	Autumn (Sep–Nov)	5	3037	40	59.9	< 0.01	93.3%	1.32% (0.91–1.73)
	Winter (Jan–Feb, Dec)	5	3994	147	2.6	0.11	–	3.68% (3.10–4.26)
Case-type	Outpatient	3	4852	156	0.7	0.39	–	3.22% (2.72–3.72)
	Inpatient	3	12,912	460	0.2	0.66	–	3.56% (3.24–3.88)

The epidemiological results of 17 studies showed that there is no significant relationship between gender and HMPV infection, the average positive rate of HMPV infection among male and female was 3.48% (95% CI 3.33–3.62) and 5.11% (95% CI 4.88–5.34). Moreover, the positive rate of HMPV-infected inpatients (3.56%, 95% CI 3.24–3.88) was not significantly different between the outpatients (3.22%, 95% CI 2.72–3.72).

#### The genetic features of HMPV in China

According to the genetic characteristics of HMPV, which could be divided into six lineages (A1, A2a, A2b, A2c, B1, and B2) [103, 104]. Though the relationships between pathogenicity and different HMPV lineages have been not fully explored, it is necessary to monitor the geographical distribution of different HMPV lineages in China. In this review, a total of 1253 HMPV gene sequences (G, F, M, or complete genome) were summarized in 19 articles to explore the genetic features of HMPV strains prevalent in China. As shown in Table 3, 8, 35, 544, 85, 299, and 282 HMPV strains belonged to the A1, A2, A2b, A2C, B1, and B2 lineages, which accounted for 0.64%, 2.79%, 43.42%, 6.78%, 23.86%, and 22.51%, respectively. The summarized results indicated that the A2b, B1, and B2 strains are the predominant lineages prevalent in China, while HMPV lineages prevalent in different provinces/regions in China are diverse. For example, the A2C was the predominant lineage prevalent in Henan province, while B1 and B2, but not A2b or A2C, predominated in Zhejiang province (Table 3).

**Table 3:** The lineage and its proportion of HMPV strains prevalent in different regions/provinces in China

Regions	No. Of studies	HMPV Lineages						Total
		A1	A2	A2B	A2C	B1	B2	
Henan province	1			1	31	5	6	43
Beijing city	7	1		340		140	188	669
Hainan province	1			4			2	6
Zhejiang province	1	6				12	10	28
Hubei province	1	1		61		42	41	145
Hunan province	3			134	1	59	14	208
Jiangxi province	1		21			3	4	28
Guangdong province	1			3		2	1	6
Tianjin city	1		14			1	3	18
Southern China	1			1	17	27	9	54
Beijing, Liaoning, Gansu, Guangdong, Zhejiang, and Guizhou provinces	1				36	8	4	48
Total	19	8	35	544	85	299	282	1253

## Conclusion and further directions

HMPV is still one of the major infectious viruses that cause ARTIs today, and it has been continuously posing huge economic burdens on the World Public Health Organization. Despite numerous efforts focused to develop diagnostic technologies for HMPV detection, epidemiological surveys, vaccines and other antiviral agents against this pathogen, there are currently no effective vaccines or medications against HMPV infection [15]. In light of this, rapid identification of positive cases and comprehensive investigation of HMPV epidemiological features are essential to prevent HMPV outbreaks and lessen their harmful effects.

There are currently many nucleic acid-based technologies for molecular HMPV detection available, which significantly lessen the challenge of HMPV diagnosis in various diagnostic contexts [22–24, 26–28, 34, 35, 38, 45, 48]. Among these, RT-PCR has been extensively used for pathogen detection, while this method is not appropriate for early HMPV diagnosis because of its low sensitivity [22–24]. The RT-qPCR method has a higher sensitivity than the traditional RT-PCR, and requires less operation time and fewer equipment, thus RT-qPCR has been widely used for the timely molecular detection of HMPV [26]. Additionally, a number of multiple RT-qPCR techniques have been developed and are used to simultaneously detect different pathogens including HMPV, in light of the relatively low prevalence of HMPV compared to other ARTIs-related viruses and the frequent occurrence of co-infection[27, 28]. Compared with the single RT-qPCR, multiple RT-qPCR methods are less costly and less time-consuming [27, 28].

Moreover, visual detection methods such as LAMP [34], RAA [38], and CRISPR/Cas12a combined with RPA [45], have also been developed for HMPV detection. These detection methods exhibit high sensitivity and do not require complex or expensive equipment, making them alternative approaches for HMPV detection in certain special occasions, such as field inspections at home or customs centers. Furthermore, mNGS is an emerging molecular detection technology that can directly identify a large number of pathogenic microorganisms in clinical samples, as well as amplify the complete genome of causative agents [105, 106]. While mNGS is time-consuming and more expensive compared to other molecular detection technologies, it may not be suitable for severely infected patients [48]. Thus, each molecular detection method has its own advantages and disadvantages. These characteristics should be fully considered in the clinical application.

In terms of the epidemiological characteristics of HMPV in China, the documents obtained from English and Chinese databases showed that HMPV has been widely prevalent in different regions of China. Although the prevalence of HMPV is relatively lower than some ARTIs-related viruses, such as HRV and RSV, the threats of it poses to public health cannot be ignored [107]. Overall, the pooled molecular prevalence of HMPV was 4.70% (95% CI 4.61–4.80), as supported by a nationwide prospective surveillance survey conducted in China, which showed a 4.1% HMPV-positive rate among 231,107 eligible patients with acute respiratory infection from 2009 to 2019 [107]. Notably, the prevalence of HMPV varied significantly by geographical region. This variation may be attributed to the differences in molecular detection methods, sampling sizes, patients ages, and other factors, all of which require further investigation.

The results of additional analysis revealed a significant association between the occurrence of HMPV infection and sampling season as well as patients age. In terms of sampling seasons, patients with ARTIs in spring had nearly 9 times the risk of HMPV infection compared to those in summer and autumn. As for age, the prevalence of HMPV among patients with ARTIs was significantly higher in young children (< 60 months) compared to older patients (> 60 months). Young children are at a high risk of HMPV infection in spring, while the threat of this pathogen to elderly people in other seasons should not be neglected. However, the gender and disease severity (inpatients or outpatients) had limited effects on the rates of HMPV infection among patients in China.

Due to the significant genetic variation of HMPV strains, they can be divided into multiple genotypes (A1, A2, B1, B2) and lineages (A1, A2a, A2b, A2c, B1, B2) [10, 11]. It is important to note that the genotypes or lineages of HMPV may vary from one region to another [5, 108]. Our summarized data show that at least five HMPV lineages (A1, A2b, A2C, B1, and B2) have been prevalent in China, while the lineages and proportions of HMPV vary greatly from province to province or region to region. Overall, the A2b, B1, and B2 lineages have become the predominant strains in China, while the A1 lineage was seldom detected in the regions under investigation. However, several characteristics of different HMPV lineages have not been comprehensively uncovered, including virulence, pathogenicity, and clinical symptoms. These issues need to be addressed in the future.

It is worth noting that the epidemiological characteristics of HMPV in China were not comprehensively analyzed in this review, and several factors contributed to this. Firstly, the research scope in this review may not cover all available reports. Secondly, although 56 representative studies were included in this review to assess the prevalence of HMPV in China, essential background information such as sampling season and year, disease severity level (inpatients or outpatients), viral load of the tested sample, and other risk factors were not provided in some of the papers. Thirdly, most of the HMPV epidemiological research has been reported in Guangdong, Jiangsu, and Beijing. The epidemiological characteristics of HMPV in other regions or provinces were relatively limited, and even in deficiency. Fourthly, this review summarized the types and proportions of different HMPV lineages in the investigated regions. However, most of the papers did not provide essential information on viral load, clinical signs, or disease severity of patients infected with different HMPV lineages. These limitations hinder a comprehensive evaluation of the epidemiological features of HMPV in China. Therefore, it is suggested that future HMPV epidemiological survey should overcome these potential drawbacks.

In summary, a variety of molecular diagnostic methods have been developed for detecting HMPV in China. RT-PCR, RT-qPCR, and multiple RT-qPCR are the most widely used approaches for clinical HMPV detection. As alternative options, other visual detection methods should be implemented in clinical testing as early as possible, with the consideration of lower cost, higher sensitivity, and higher convenience. The summarized data showed a relatively low prevalence (4.70%) of HMPV infection among patients with ARTIs in China. The prevalence was significantly associated with sampling seasons and patients' ages. Moreover, multiple HMPV lineages are prevalent in China, with high proportions of the A2b, B1, and B2 lineages. Given the persistent threat of HMPV to public health [109], it is crucial to continue monitoring the epidemiological spread and genetic evolution characteristics of this pathogen in China.

### Supplementary Information

**Additional file 1: **Detailed information of 56 research investigating the epidemiological characteristics of HMPV in China.

## Data Availability

The data presented in this review can be found in online repositories.

## Abbreviations

ARTIs: Acute respiratory tract infections
LRTIs: Lower respiratory tract infections
HRSV: Human respiratory syncytial virus
HMPV: Human metapneumovirus
RT-PCR: Reverse transcription polymerase chain reaction
RT-qPCR: Real-time quantitative reverse transcription polymerase chain reaction
RT-LAMP: Reverse transcription loop-mediated isothermal amplification
GeXP: GenomeLab Gene Expression Profiler genetic analysis system
HRV: Human rhinovirus
DMF: Digital microfluidic
LOD: Limit of detection
RAA: Recombinase-aided amplification
CRISPR: Clustered regularly interspaced short palindromic repeat
Cas: CRISPR-associated proteins
RPA: Recombinase polymerase amplification
LF: Lateral flow
mNGS: Metagenomic next-generation sequencing
