# Supplementary material for: Epidemiology and diagnosis technologies of human metapneumovirus in China: a mini review
Source: Virol J. 2024 Mar 7;21:59. doi: 10.1186/s12985-024-02327-9 (PMC10921660; doi:10.1186/s12985-024-02327-9)
Supplement: Supplementary file 1 — Additional file 1: Detailed information of 56 research investigating the epidemiological characteristics of HMPV in China. [file 12985_2024_2327_MOESM1_ESM.docx]

**Supplementary Table 1**: Prime sequences of the difference HMPV molecular diagnosis methods included in this review

| Methods (targeted gene) | Primer | Sequences (5’-3’) | Reference |
| --- | --- | --- | --- |
| Nested PCR (N gene) | Out forward primer | TTAARTTACAAAAAAACATGGGAC | [23] |
|  | Out reverse primer | AAAGAATATCTTTTCCTTCAGGG |  |
|  | Internal forward primer | ATGGGACAAGTGAAAATGTCTC |  |
|  | Internal reverse primer | AATTACTCATAATCATTTTGACTG |  |
| Nested PCR (F gene) | Out forward primer | CAATGCAGGTATAACACCAGCAATATC | [22] |
|  | Out reverse primer | GCAACAATTGAACTGATCTTCAGGAAAC |  |
|  | Internal forward primer | ACATGCCAACATCTGCAGGACAAATAAAAC |  |
|  | Internal reverse primer | ACATGCTGTTCACCTTCAACTTTGC |  |
| GeXP-based multiplex RT-PCR (HMPV, L and N genes) | HMPV-LF1 | AGGTGACACTATAGAATACATGCCCACTATAAAAGGTCAG | [24] |
|  | HMPV-LR1 | GTACGACTCACTATAGGGACACCCCAGTCTTTCTTGAAA |  |
|  | HMPV-NF2 | AGGTGACACTATAGAATAGAGCTAAYAGAGTGCTAAGTGATG |  |
|  | HMPV-NR2 | GTACGACTCACTATAGGGAACTTTCTGCTTTGCTTCCTGT |  |
| RT-PCR (F gene) | Forward primer | CTTTGGACTTAATGACAGATG | [25] |
|  | Reverse primer | GTCTTCCTGTGCTAACTTTG |  |
| RT-qPCR (F gene) | Forward primer | TTCGGAATCTGATAGGGGTCT |  |
|  | Reverse primer | CCTGTGCTAACTTTGCATGGGT |  |
|  | Probe | CTCCGTAATTTACATGGTGCAGCTGCC |  |
| RT-qPCR (N gene) | Forward primer | CATCAGGTAATATCCCACAAAATCAG | [26] |
|  | Reverse primer | GTGAATATTAAGGCACCTACACTAATAA |  |
|  | Probe | TCAGCACCAGACACAC |  |
| RT-qPCR (L gene) | Forward primer | TGCTCATGCCCACTATAAAGGGT | [26] |
|  | Reverse primer | TCTGTTAATATCCCACACCAATGAC |  |
|  | Probe | TATCAGCAGCATTAGCA |  |
| RT-qPCR (N gene) | Forward primer | CTGTTTGTGAACATTTTYATGCA | [27] |
|  | Reverse primer | ACAGAGACATGGCCTAACATDAT |  |
| One-step triplex RT-qPCR assay (HMPV, F gene) | Forward primer | TCAGAATGCAGGGTCAACTGTT | [28] |
|  | Reverse primer | GACATGGTCTCCTCTTGTTTCACA |  |
|  | Probe | FAM-CAAGCTTCCCGTTCTCAGCC-MGBNFQ |  |
| Digital microfluidic RT-qPCR  (HMPV, F gene) | Forward primer | CTGTCAGCTTCAGTCARTTCAAC | [32] |
|  | Reverse primer | CAATGATATTGCYGGTGTTAT |  |
|  | Probe | FAM-TGTTGTGCGGCAGTTTTCAGACAAT-BHQ1 |  |
| RT-LAMP (N gene) | F3 | ACAGGAGTCTATTCATTGAGT | [35] |
|  | B3 | ACCAAATCATAAACCTCTGTG |  |
|  | FIP (F1c+F2) | CGGCTCCATAAGCTTGCATAAATAT-GGGAAAGCTTTAGGCTCA |  |
|  | BIP (B1c+B2) | ACAATGCTAAGGTGGGGTGTC-CTTCAATTCAGCTTGCACAG |  |
|  | LF | CAAACAAACTTTCTGCTTTGCTTCC |  |
|  | LB | CATCTAACAACATAATGCTAGGGCA |  |
| RT-LAMP (HMPV genotype A, M gene) | A-F3 | TTCAGGCCAATACACCAC | [34] |
|  | A-B3 | GTCAAGTGCTACAGTCGC |  |
|  | A-FIP | ACTTTGTGATGCAGCATACAGA-TTTTCAGTTCTGCTTGATCAGCT |  |
|  | A-BIP | ACTAAAAGTGAATGCATCAGCYC-TTTTACTTCAAACYTTTTGGGAAG |  |
| RT-LAMP (HMPV genotype B, M gene) | B-F3 | GTGTCAAAATTTGTGAGTTCAG | [34] |
|  | B-B3 | GGCYTCRCTGCTTATTGC |  |
|  | B-FIP | TTCTCTAGGTCCATGAAGTCACA-TTTTCAAATCAGTTGGCAAAAAGACA |  |
|  | B-BIP | TACCTGTGACAATACCAGCATTC-TTTTCAGTRGCTGACTCACTCTCT |  |
| RT-RAA | Forward primer | AGTCCAGGTGGTTCTAACCAAAACATACTCC | [38] |
|  | Reverse primer | TTGTGGGATGTTACCTGATGACTCTTTAAGC |  |
|  | Probe | GGGAAGAGCTGCAGAT(FAM)(THF)T(BHQ)TAGATATACATGGAGTGGAAAAGAGTTGGG |  |
| RT-RPA (N gene) | N-F4 | GGCATGTATCTGTGCAAGCTGA | [45] |
|  | N-R4 | TCCCTCTGTACATTCCTATT |  |
| Cas12a detection (RT-RPA amplication production) | CrRNA-F | GAAATTAATACGACTCACTATAGGGTAATTTCTACTAAGTGTAGATAGGCAAAGCCCAAAAGCTGG |  |
|  | CrRNA-R | CCAGCTTTTGGGCTTTGCCTATCTACACTTAGTAGAAATTACCCTATAGTGAGTCGTATTAATTTC |  |
|  | SsDNA reporter-F | 6-FAM-TTATTATT-BHQ1 |  |
|  | SsDNA reporter-R | 6-FAM-TTATTATT-Biotin |  |

**Supplementary Table 2**: Detailed information of 56 research investigating the epidemiological characteristics of HMPV in China

| **Number** | **Author** | **Province/region** | **Time** | **Publication time** | **Approach** | **Positive rate** | **Genotype** | **References** |
| --- | --- | --- | --- | --- | --- | --- | --- | --- |
| 1 | Zhu et al | Liaoning, Jilin, Ningxia, Hebei, Zhejiang, Shanghai, Guangdong, Chongqing | 2014.12-2016.6 | 2021 | Multiple PCR | 7.8% (213/2721) |  | [53] |
| 2 | Zhao et al | Beijing, Liaoning, Gansu, Guangdong, Zhejiang, and Guizhou | 2017.12-2019.12 | 2022 | Luminex xTAG respiratory viral panel assay | 5.3% (145/2773) | A2c (75.0%, 36/48), B1 (16.67%, 8/48), and 8.33% (4/48) | [54] |
| 3 | Tang et al | Suzhou city of Jiangsu province | 2018.3-2019.12 | 2022 | RT-PCR | 2.11% (105/4980) |  | [7] |
| 4 | Tang et al | Suzhou city of Jiangsu province | 2017.3-2021.10 | 2023 | RT-PCR | 2.21% (189/8430) |  | [55] |
| 5 | Li et al | Shenzheng city of Zhejiang province | 2019-2020 | 2021 | Multiple PCR | 4.55% (259/5696) |  | [56] |
| 6 | Xie et al | Luohe city of Henan province | 2017-2019 | 2022 | RT-PCR | 7.1% (72/1021) | A2b (n=1), A2c (n=31),  B1 (n=5), and B2 (n=6) | [57] |
| 7 | Zhu et al | Beijing | 2010.8-2016.7 | 2022 | RT-PCR | 2.7% (292/10918) | A2b (47.9%, 139/290),  B1 (23.4%, 68/290), and  B2 (28.3%, 82/290) | [58] |
| 8 | Wang et al | Beijing | 2017.4-2018.3 | 2021 | RT-PCR | 4.08% (52/1276) | A2b (39.6%, 19/48), B1 (54.2%, 26/48), and B2 (6.3%, 3/48) | [59] |
| 9 | Li et al | Shanghai | 2010-2020 | 2022 | DFA assay | 0.7% (41/5544) |  | [60] |
| 10 | Ye et al | Southern China | 2010-2019 | 2023 | Real-time PCR | 3.5% (524/14817) | A2b (1.9%), A2c (31.5%), B1 (50.0%), and B2 (16.7%) | [9] |
| 11 | Li et al | Shanghai | 2017.4-2018.3 | 2021 | RT-qPCR | 1.5% (6/397) |  | [60] |
| 12 | Gong et al | Beijing | 2018.11-2021.8 | 2022 | RT-qPCR | 7.9% (124/1572) | A2b (82.1%, 67/78), B1 (11.5%, 9/78), and B2 (6.4%, 5/78) | [22] |
| 13 | Cui et al | Anhui, Beijing, Guangdong, Hebei, Hunan, Jilin, Shandong, Shaanxi, Xinjiang | 2009-2021 | 2022 | RT-qPCR | 4.12% (477/11591) |  | [61] |
| 14 | Du et al | Hangzhou city of Zhejiang province | 2020.12-2021.3 | 2022 | RT-qPCR | 7.14% (103/1442) | B1 (1.6%, 1/61) and B2 (98.4%, 60/61) | [62] |
| 15 | Mai et al | Hainan province | 2019-2021 | 2022 | Multiple PCR | 2.88% (7/243) | A2b (66.67%, 4/6) and B2 (33.33%, 2/6) | [63] |
| 16 | Ji et al | Huzhou city of Zhejiang province | 2016.1-2020.12 | 2021 | RT-qPCR | 4.94% (56/1133) | A1 (21.43%, 6/28), B1 (42.86%, 12/28), and B2 (35.71%, 10/28) | [64] |
| 17 | Shi and Huang | Suzhou city of Jiangsu province | 2021.9-2022.12 | 2023 | Multiple PCR | 10.5% (1092/10396) |  | [65] |
| 18 | Zhang et al | Shandong province | 2019.3-2021.12 | 2023 | A multiplex panel assay | 4.18% (140/3348) |  | [66] |
| 19 | Chan et al | Hongkong | 2014.3-2023.4 | 2023 | Multiple PCR | 3.71% (747/20127) |  | [67] |
| 20 | Yi et al | Guangdong province | 2013.1-2017.12 | 2019 | Real-time PCR | 5.2% (76/1460) |  | [68] |
| 21 | Liu et al | Guangzhou city of Guangdong province | 2009.7-2016.6 | 2019 | Real-time PCR | 2.8% (321/11398) |  | [69] |
| 22 | Zhao et al | Beijing and Shanghai | 2008.5-2014.3 | 2019 | Real-time PCR | 5.0% (35/700) |  | [70] |
| 23 | Li et al | Guangzhou city of Guangdong province | 2015.5-2018.4 | 2019 | Real-time PCR | 2.1% (14/659) |  | [71] |
| 24 | Zhong et al | Zhejiang province | 2013.12-2015.6 | 2019 | Real-time PCR | 6.97% (93/1335) |  | [72] |
| 25 | Li et al | Gansu province | 2011.1-2015.12 | 2018 | RT-PCR | 1.6% (45/2768) |  | [73] |
| 26 | Zhou et al | Changsha of Hunan province | 2011.4-2013.3 | 2017 | Real-time PCR | 18.2% (199/1092) |  | [74] |
| 27 | Ye et al | Shanghai | 2012-2015 | 2016 | RT-PCR | 2.07% (20/967) |  | [75] |
| 28 | Yan et al | Lanzhou city of Gansu province | 2011.12-2012.12 | 2017 | Real-time PCR | 12.14% (47/387) |  | [76] |
| 29 | Kong et al | Wuhan city of Hubei province | 2008.7-2013.12 | 2016 | Real-time PCR | 4.40% (171/3883) | A1 (0.69%, 1/145), A2 (42.07%, 61/145), B1 (28.97%, 42/145), and B2 (28.28%, 41/145) | [77] |
| 30 | Wang et al | Suzhou city of Jiangsu province | 2011-2014 | 2016 | RT-PCR | 3.5% (128/3662) |  | [78] |
| 31 | Zeng et al | Changsha of Hunan province | 2007.9-2011.2 | 2015 | Real-time PCR | 5.2% (135/2613) | A2b (72.6%, 98/135), B1 (26.6%, 36/135), and B2 (0.7%, 1/135) | [79] |
| 32 | Luo et al | Yangshan county of Zhejiang province | 2012-2014 | 2016 | Multiple PCR | 10.86% (26/240) |  | [80] |
| 33 | Tian  et al | Guiyang city of Guizhou province | 2020.7-2021.1 | 2021 | Real-time PCR | 6.1% （16/262） |  | [81] |
| 34 | Liu et al | Jiangxi province | 2010-2016 | 2019 | RT-PCR | 2.21 (54/2438) | A2 (75.0%, 21/28), B1 (10.71%, 3/28), and B2 (14.29%, 4/28) | [82] |
| 35 | Liu et al | Shenzhen city of Guangdong province | 2015.6-2016.6 | 2018 | RT-PCR | 21.05% (362/1720) | A2b (50.0%, 3/6), B1 (33.33%, 2/6), and B2 (16.67%, 1/6) | [83] |
| 36 | Peng et al | Yueyang city of Hunan province | 2019.7-2020.6 | 2023 | Real-time PCR | 1.9% (60/3175) | A2B (53.3%, 32/6), B1 (31.7%, 19/60), and B2 (15.0%, 9/60) | [84] |
| 37 | Wei et al | Wuhan city of Hubei province | 2021.6-2022.2 | 2023 | Multiple PCR | 1.6% (54/3365) |  | [85] |
| 38 | Malik et al | Hongkong | 2001.8-2002.3 | 2003 | RT-PCR | 5.5% (32/587) |  | [86] |
| 39 | Chan et al | Hongkong | Before 2003.3 | 2003 | RT-PCR | 52.1% (25/48) |  | [87] |
| 40 | Lee et al | Hongkong | 2003.4-6 | 2007 | Nested RT-PCR | 20.0% (31/155) |  | [88] |
| 41 | Mao et al | Chongqing | 2004.12-2005.7 | 2008 | Virus isolation | 6.98% (6/86) |  | [89] |
| 42 | Liu et al | Hunan province | 2005 | 2008 | RT-PCR | 7.3% (17/232) | A1(1), A2 (4), B1 (4), and B2(4) | [90] |
| 43 | Li et al | Tianjin | 2006.5-2009.1 | 2009 | Nest RT-PCR | 6.5% (20/310) | A2 (70%, 14/18), B1 (5%, 1/18) and B2 (15%, 3/18) | [91] |
| 44 | Ji et al | Suzhou of Jiangsu province | 2005.12-2006.3 | 2009 | RT-PCR | 6.6% (128/1932) |  | [92] |
| 45 | Zou et al | Guangzhou of Guangdong province | 2006.10-2008.8 | 2009 | RT-PCR | 7.49% (39/521) |  | [93] |
| 46 | Ren et al | Beijing | 2005.5-2007.7 | 2009 | RT-PCR | 0.3% (19/5808) |  | [94] |
| 47 | Chen et al | Chongqing | 2006.4-2008.3 | 2010 | RT-PCR | 25.9% (227/878) | A2 (13/14) and B2 (1/14) | [95] |
| 48 | Xiao et al | Gansu province | 2006.11-2008.12 | 2010 | RT-PCR | 6.80% (45/661) | A2 (80%), A1 (2.2) and  B1 (17.8%) | [96] |
| 49 | Li et al | Beijing | 2008.7-2010.6 | 2012 | RT-PCR | 1.7% (49/2936) | A2b (28.6%), B1 (38.8%), and B2 (32.6%) | [97] |
| 50 | Jin et al | Lanzhou of Gansu province | 2006-2009 | 2012 | RT-PCR | 6.15% (50/813) |  | [98] |
| 51 | Lu et al | Beijing | 2008.7-2010.6 | 2013 | RT-PCR | 6.3% (65/1028) | A2 (61.58%, 40/65), B1 (12.31%, 8/65), B2 (26.15%, 17/65) | [99] |
| 52 | Wang et al | Suzhou of Jiangsu province | 2006.1-2009.12 | 2013 | RT-PCR | 8.9% (596/6655) |  | [100] |
| 53 | Zhang et al | Chongqing | 2008.4-2011.3 | 2012 | RT-PCR | 10.2% (144/1410) | A1 (1/11), B1 (1/11), A2b (9/11) | [101] |
| 54 | Lu et al | Jinan of Shandong province | 2019.7-2020.6 | 2013 | RT-PCR | 5.57% (18/323) |  | [102] |
| 55 | Feng et al | China | 2009-2013 | 2014 | Multiple RT-PCR | 1.5% (424/10387) |  | [103] |
| 56 | Wei et al | Beijing | 2017-2019 | 2023 | RT-qPCR | 6.18% (176/2828) | A2b (92.57%, 25/27), B1 (6.43%, 2/27) | [104] |
